# Aberrant Bcl-x splicing in cancer: from molecular mechanism to therapeutic modulation

**DOI:** 10.1186/s13046-021-02001-w

**Published:** 2021-06-12

**Authors:** Zhihui Dou, Dapeng Zhao, Xiaohua Chen, Caipeng Xu, Xiaodong Jin, Xuetian Zhang, Yupei Wang, Xiaodong Xie, Qiang Li, Cuixia Di, Hong Zhang

**Affiliations:** 1 Department of Heavy Ion Radiation Medicine, Bio-Medical Research Center, Institute of Modern Physics, Chinese Academy of Sciences, Lanzhou, 730000 China; 2 Key Laboratory of Heavy Ion Radiation Biology and Medicine of Chinese Academy of Sciences, Lanzhou, 730000 China; 3 College of Life Sciences, University of Chinese Academy of Sciences, Beijing, 101408 China; 4 School of Nuclear Science and Technology, University of Chinese Academy of Sciences, Beijing, 101408 China; 5 Medical Genetics Center of Gansu Maternal and Child Health Care Center, Lanzhou, 730000 China; 6 School of Basic Medical Sciences, Lanzhou University, Lanzhou, 730000 China; 7 Advanced Energy Science and Technology Guangdong Laboratory, Huizhou, 516029 China

**Keywords:** Bcl-x, Alternative splicing, Cell apoptosis, Splicing correction, Splice-switching oligonucleotides, Small molecular modulators

## Abstract

Bcl-x pre-mRNA splicing serves as a typical example to study the impact of alternative splicing in the modulation of cell death. Dysregulation of Bcl-x apoptotic isoforms caused by precarious equilibrium splicing is implicated in genesis and development of multiple human diseases, especially cancers. Exploring the mechanism of Bcl-x splicing and regulation has provided insight into the development of drugs that could contribute to sensitivity of cancer cells to death. On this basis, we review the multiple splicing patterns and structural characteristics of Bcl-x. Additionally, we outline the cis-regulatory elements, trans-acting factors as well as epigenetic modifications involved in the splicing regulation of Bcl-x. Furthermore, this review highlights aberrant splicing of Bcl-x involved in apoptosis evade, autophagy, metastasis, and therapy resistance of various cancer cells. Last, emphasis is given to the clinical role of targeting Bcl-x splicing correction in human cancer based on the splice-switching oligonucleotides, small molecular modulators and BH3 mimetics. Thus, it is highlighting significance of aberrant splicing isoforms of Bcl-x as targets for cancer therapy.

## Background

Apoptosis regulator Bcl-extra (Bcl-x), also named BCL2L or BCL2L1, is a typical example of apoptotic response gene impacted by splicing. It is an essential member of B-cell lymphoma 2 (Bcl-2) apoptosis family that regulates cell fate [1, 2]. Bcl-x nascent transcripts are alternatively spliced and mainly encode two antagonistic isoforms. The long isoform Bcl-xL blocks apoptosis by inhibiting pro-apoptotic counterparts of Bcl-2 family, whereas the short isoform Bcl-xS can promote apoptosis [2]. An increasing body of data suggests that dysregulated expression of Bcl-x apoptotic isoforms contributes to multiple hallmarks of human cancers. For example, Bcl-xL level was strongly enhanced in cancer cells at the invasive forefront of human breast carcinomas and simultaneously acquired resistance to apoptotic stimuli [3, 4]. However, Bcl-xS conferred the therapeutic sensitivity by decreasing the apoptosis threshold [5]. The ratio of pro-apoptotic Bcl-xS and anti-apoptotic Bcl-xL proteins plays a vital role in regulating the switch between cell life and death. Hence, in this review, we summarize the patterns and the splicing regulatory network of Bcl-x pre-mRNA splicing. In addition, we describe how this aberrant splicing impacted apoptosis, autophagy, invasion and metastasis, immune response, as well as clinical therapy resistance in cancer. Furthermore, we outline the emerging strategies that modulate the cancerous Bcl-x splicing and restore the balance of Bcl-xL/Bcl-xS ratio. Targeting the oncogenic splicing of Bcl-x is believed to result in sensitized cell death by simultaneously blocking Bcl-xL and enhancing Bcl-xS splicing [6].

### Splicing isoforms of Bcl-x

**Fig. 1:**
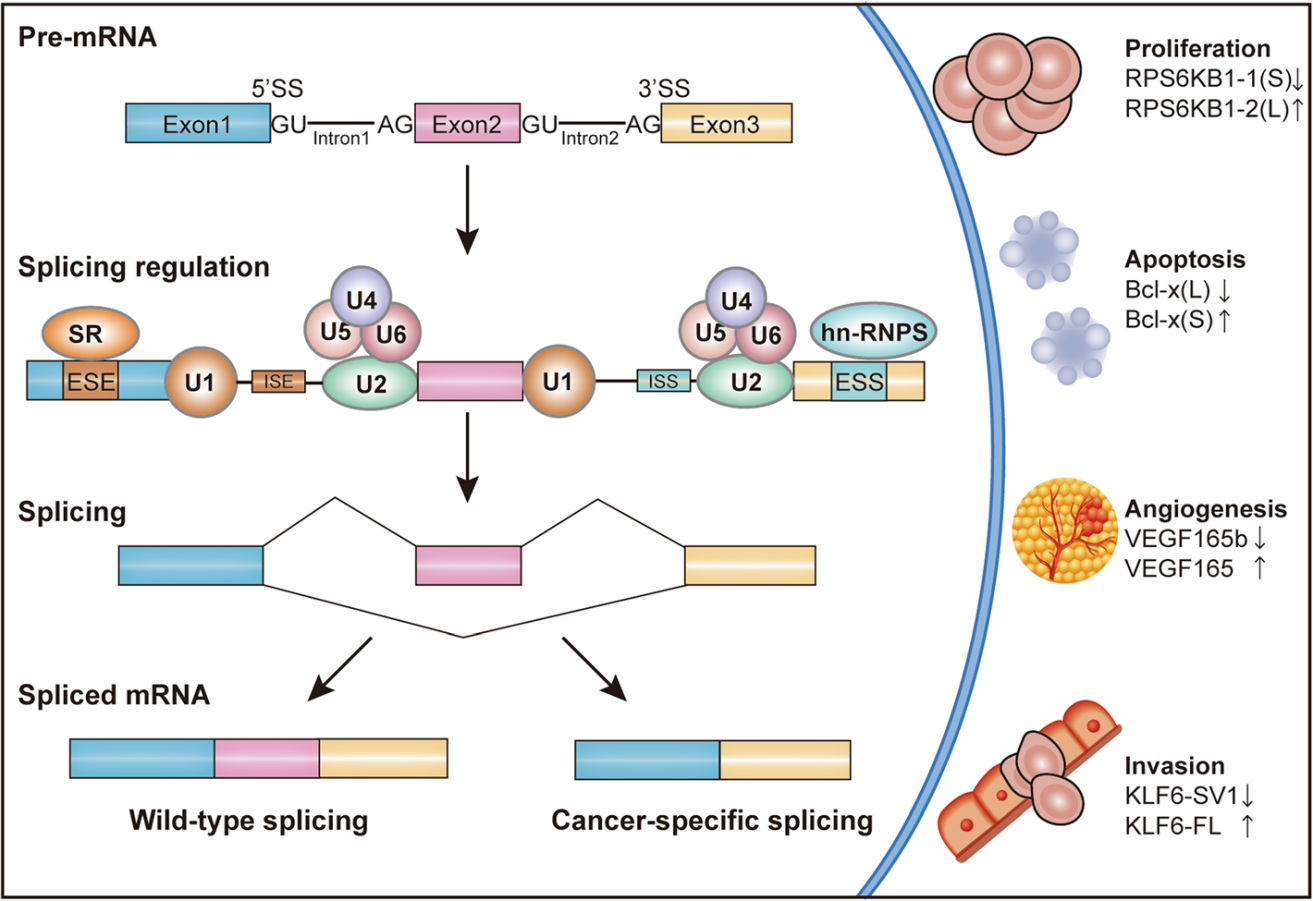
Alternative splicing and the effect of aberrant alternative splicing on cancer progression. The spliceosome, consists of five small nuclear ribonucleoproteins particles (U1, U2, U4, U5 and U6) and hundreds of additional proteins, recognizes the consensus sequence of each intron and assembles reversibly on splice sites to catalytic pre-mRNA splicing. SR  proteins  and hnRNPs bound to exonic or intronic regulatory elements to promote or prevent the use of splice sites thus affecting alternative splicing decisions. The figure displays some examples of cancer-specific splicing events that contribute to distinct hallmarks of cancer. Arrows up and down indicate the corresponding isoforms contributing or suppressing the hallmark respectively

Alternative splicing expands the coding capacity of genomes of eukaryotes significantly through splice site selection (Fig. 1) [7]. Nearly >95% of human multi-exonic genes could be alternatively spliced into mRNAs isoforms [8]. Splicing reaction is orchestrated by a highly dynamic ribonucleoprotein complex known as spliceosome and hundreds of related proteins [9]. The spliceosome recognizes and assembles reversibly on pre-mRNA to catalytic splicing in a stepwise manner. This process is further modulated by a number of cis-acting elements and trans-acting factors (splice factors) bound to them [9]. Indeed, mutations in cis-regulatory sequences and spliceosomal associated proteins are enriched in cancer. These mutations always affect the splicing of cancer-related genes [10, 11]. A growing body of evidence has revealed aberrant splicing events as contributors of hallmarks of tumorigenesis, such as proliferation, angiogenesis, invasion and apoptosis (Fig. 1) [11, 12].

**Fig. 2:**
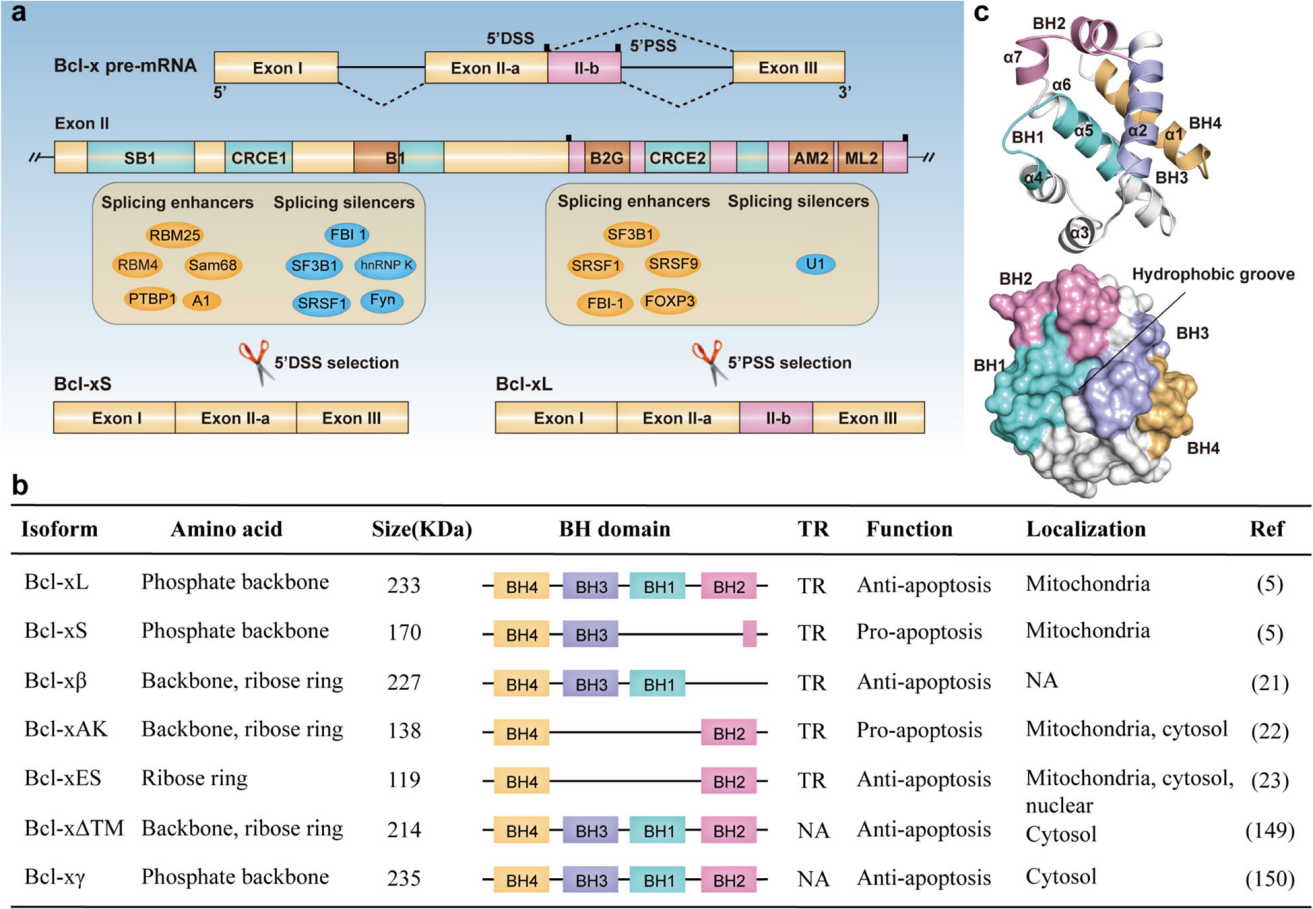
Bcl-X pre-mRNA splicing and structures of splicing isoforms. **a**. Alternative splicing mode and splicing regulation. Splicing occurred closer to the 5'PSS of exon 2 produces the long isoform Bcl-xL. Alternative splicing occurred near the 5' DSS of exon 2 produces the short isoform Bcl-xS. In addition, distinct cis-elements and splice factors bind to cis-elements to influence the alternative 5' splice site selection of Bcl-x pre-mRNA. **b**. General characteristics of isoforms spliced from Bcl-x pre-mRNA. **c**. The protein structures of Bcl-xL. The secondary structure of Bcl-xL and the position in the space of BH domains (up). Tertiary structure of Bcl-xL and the BH domain and hydrophobic groove are showed (down)

Bcl-x, a critical apoptotic gene of the Bcl-2 family, is located in chromosome 20 (20q11.1). It was first discovered by using Bcl-2 fluorescent probe hybridization in chickens [2]. Subsequently, two antagonistic isoforms of Bcl-x pre-mRNA in human body were isolated, which then were identified as Bcl-xL and Bcl-xS, respectively. Splicing selection closer to the proximal 5' splice site (5'PSS) of exon 2 resulted in the long anti-apoptosis isoform Bcl-xL (Fig. 2a), which contained four exons and was composed of 780 bp. The primary structure of Bcl-xL is composed of 233 aa, which contains C-terminal hydrophobic transmembrane (TM) domain responsible for the anchoring to membranes and all four BH domains (BH1-4) (Fig. 2b,c). When the splicing occurred near the cryptic distal 5' splice site (5' DSS) of exon 2, the short isoform pro-apoptotic Bcl-xS with 591 bp was produced. Stable Bcl-xS expression played an important role in regulating the ability of pro-survival genes to inhibit apoptotic cell death [2]. Bcl-xS protein is encoded by 170 aa containing both BH3, BH4, and TM domains but lacking BH1 and BH2 domains, which might lead to alternation of hydrophobicity. In addition, other splice isoforms encoded by Bcl-x also had been identified in different cell types and performed diverse functions (Fig. 2b) [13–17]. Despite multiple isoforms that Bcl-x could splice, the pro-apoptotic Bcl-xS and anti-apoptotic Bcl-xL are still the predominant isoforms acting in cell fate.

### Regulation of Bcl-x pre-mRNA splicing

#### Cis-regulatory elements

Cis-regulatory elements are short nucleotide motifs within pre-mRNA transcripts that providing binding sites for specific trans-acting factors. It can be categorized into exonic splicing enhancers (ESEs), exonic splicing silencers (ESSs), intronic splicing enhancers (ISEs) and intronic splicing silencers (ISSs) depending on their position and impact on the use of splice site [18] Bcl-x pre-mRNA contains several cis-elements acting as splicing activators or repressors by interacting with related splicing factors (Fig. 2a). For example, SB1 (361bp), located in the first half of exon 2, was defined as an ESE because splicing to Bcl-xS was even stronger in the absence of SB1. Similarly, DNA damage-induced Bcl-xS splicing only increased in the presence of SB1 [19]. In addition, B1 was a composite element located upstream of 5' DSS of Bcl-x pre-mRNA. The 5' portion of B1 displayed ESE activity, whereas the 3' portion was occupied with ESS element. hnRNP K bound to the silencer portion of B1 to repress the production of Bcl-xS [20]. Moreover, B2G module was a 30-nucleotides G-rich element located immediately downstream of the 5' DSS. Combination of hnRNP F/H to B2G enhanced Bcl-xS splicing. B3 located up-stream of 5'PSS to favor the production of Bcl-xL. The two elements ML2 and AM2 within B3 were identified to enhance Bcl-xL splicing through interacting with SRp30c [21]. Deleting B2G and B3 regions completely abrogated production of Bcl-xS and Bcl-xL respectively [22]. Another two cis-elements CRCE1 and CRCE2 within exon 2 were essential for ceramide-responsive Bcl-x expression. Mutation of CRCE1 or CRCE2 induced a decreased ratio of Bcl-xL/Bcl-xS [23]. In addition to exonic elements, the intron region downstream from Bcl-xL 5'PSS also had been identified to mediate signals from extracellular factors such as interleukin-6 and granulocyte-macrophage colony-stimulating factor (GM-CSF) to repress Bcl-xL splicing [24].

#### Trans-acting factors

A variety of trans-acting factors are involved in the formation of splicing regulatory network, including SR proteins, hnRNPs as well as some transcription factors (Fig. 2a) [25]. Posttranslational modifications of splicing factors would also change their binding state to cis-elements. For example, Sam68 bound to Bcl-x pre-mRNA specifically and recruited hnRNP A1 to a particular region, which caused the selection of Bcl-xS in a dose-dependent manner. This favor could be inverted by tyrosine phosphorylation of Sam68 [26]. In addition, SRSF1 was known to compete with hnRNPA1 to promote Bcl-xL splicing. However, the activity of SRSF1 itself was antagonized by splicing factors RBM4 and PTBP1 [27, 28]. Moreover, a multicomponent regulatory hub consisting of SRSF10, hnRNP A1/A2, and Sam68 was reported to activate the 5'DSS of Bcl-x in response to DNA damage [29]. Because alternative splicing is coupled to transcription, a large number of transcription factors were found to influence splicing selection. For instance, E2EF1 was suggested to increase Bcl-xS isoform through upregulating SRSF2 [30]. FBI -1 could interact with the full length or C-terminal domain of Sam68 to counteract Sam68-mediated apoptosis [31]. Moreover, TCERG1 [32], FOXP3 [33], ETS [34] as well as cellular signal pathways and other regulatory modes such as exon junction complex, G-rich sequences were all involved in Bcl-x splicing and had been summarized in Table 1 (Table 1).

**Table 1:** Trans-acting factors involved in Bcl-x splicing regulation

Regulation methods		Mechanism	Selection	Ref
RNA binding proteins	Sam68	Recruit hnRNP A1 to certain regions.	Bcl-xS	[26]
	SRSF1 (ASF/SF2)	Compete with hnRNP A1.	Bcl-xL	[26, 35]
	SRSF10	Collaborate with hnRNP A1/A2 and Sam68.	Bcl-xS	[29]
	SRSF2 (SC35)	As a direct transcriptional target of E2F1.	Bcl-xS	[30]
	SRSF3	Favor the selection of the 5' DSS.	Bcl-xS	[28]
	SRSF7	Favor the selection of the 5' DSS.	Bcl-xL	[28]
	SRSF9 (SRp30c)	Bind to ML2 and AM2.	Bcl-xL	[21]
	TRA2β	Favored selection of the 5' DSS.	Bcl-xS	[28]
	hnRNP F/H	Bind to B2G region.	Bcl-xS	[22, 36]
	PTBP1 (hnRNP I)	Bind to polypyrimidine to promote 5' DSS selection	Bcl-xS	[28]
	hnRNP A1	Interact with Sam68.	Bcl-xS	[25, 26]
	hnRNP A2/B1	Regulated by Fyn activity.	Bcl-xL	[37]
	hnRNP K	Bind to silencer element of the 5' DSS.	Bcl-xL	[20]
	RBM4	Antagonize oncogenic SRSF1.	Bcl-xS	[38]
	RBM10 (S1-1)	Block the GGGUAAG of exon 2.	Bcl-xS	[39]
	RBM11	Antagonize SRSF1.	Bcl-xS	[40]
	RBM25	Bind to CGGGCA sequence within exon 2.	Bcl-xS	[41]
Transcription factors	E2F1	Upregulate SC35 protein expression.	Bcl-xS	[30]
	FBI-1	Interact with Sam68 and affects its binding.	Bcl-xL	[31]
	TCERG1	Increase the elongation rate of RNAPII.	Bcl-xS	[32]
	FOXP3	Repress hnRNPF binding to 5'DSS.	Bcl-xL	[33]
	SAP155 (SF3B1)	Bind to CRCE 1 region.	Bcl-xL	[42]
Signal pathway	PKC signal	Through SB1 to repress the 5'DSS splicing.	Bcl-xL	[43]
	PI3K/PKC_ι_ signal	Regulate SAP155-CRCE1 complex formation.	Bcl-xL	[44]
	LPS/PRMT2 or TNF-α pathway	Interact with Sam68 and regulate its subcellular localization via its SH3 domain.	Bcl-xL	[45]
G4s and G4s ligands	G-quadruplexes (G4s)	Close to the two alternative 5'SS to compete with other RNA structures or proteins	Bcl-xS or Bcl-xL	[46, 47]
	G-quadruplex ligands (GQC05)	Stabilize G-quadruplexes.	Bcl-xS	[46]
EJC	Exon junction complex ( EJC )	RNPS1 and core EJC proteins control Bcl-x splicing through cis-acting elements SB1.	Bcl-xL	[48]

#### Epigenetic modifications

Epigenetic modifications had been suggested to interplay intricately with alternative splicing [49]. It had been reported that DNA methylation at exons and splicing sites were involved in over 20% splicing modulation by regulating the elongation rate of RNA polymerase II primarily [50]. In other studies, the dynamic histone acetylation mark of H3K4me3 nucleosome played a critical role in Mcl-1 pre-mRNA splicing [51]. Moreover, N6-methyladenosine modification was indicated to regulate splicing by co-localization with splice sites and reshaping the structure of pre-mRNA [52]. However, there were poor reports about whether and how the epigenetic modifications mentioned above affected the splicing decision of Bcl-x pre-mRNA. To date, ncRNA was the most common epigenetic regulation identified to influence Bcl-x splicing that functions as 'interactors' or 'hijackers' of splicing factors [49]. LncRNA BC200, LINC00162 as well as LncRNA-HEIH had been shown to modulate Bcl-x pre-mRNA splicing effectively [53–55]. To investigate the splicing mechanism regulated by ncRNA in-depth is necessary.

### The function of aberrant Bcl-x splicing in cancer

#### Apoptosis

Apoptosis, characterized by a series of morphological alternations including cell shrinkage, pyknosis and karyorrhexis, plasma membrane blebbing and apoptotic body formation, is a mechanism for all multicellular organisms to modulate cell life development [56]. Abnormalities in apoptosis play a crucial role in the progression of various human disease like cancer [57]. Therefore, targeting apoptotic pathways has been a mainstay for the cancer drug discovery and development. There are two commonly established apoptotic pathways in mammals: the extrinsic pathway of apoptosis mediated by the death receptor and the intrinsic pathway of apoptosis mediated by mitochondria [58]. The extrinsic apoptotic signal begins when extracellular death-inducing factors bind to its receptors (TNFR, TRAIL, FasL), recruiting adapter proteins (TRADD, FADD, caspase 8 and/or caspase 10) to form the death inducing signaling complex [59]. The intrinsic apoptotic pathway is closely regulated by the Bcl-2 family proteins. Bcl-2 proteins mainly were divided into three subgroups up to their BH domain: BH3 only proteins to initiate apoptosis (Bim, Bad, Bid, Noxa, Bmf, Hrk, Bik, Puma), pro-apoptotic proteins act as apoptotic executioner (Bax, Bak, Bok) and anti-apoptotic subfamily (Bcl-2, Bcl-xL, Bcl-W, A1, Bcl-B, Mcl-1) [60]. Initiated by internal stimuli such as DNA damage, hypoxia and oxidative stress, activated-BH3 only proteins inhibit the anti-apoptotic Bcl-2 proteins. Subsequently, activated and oligomerized Bax/Bak located in the mitochondrial outer membrane, promoting mitochondrial outer membrane permeability (MOMP), the release of cytochrome C and caspase activation [61]. Disruption in the balance of pro-apoptotic and anti-apoptotic members of the Bcl-2 family proteins promotes carcinogenesis and cancer cell survival [62]. More importantly, anti-apoptotic Bcl-2 proteins are widely over-expressed in cancers and have been established to contribute to therapy resistance, recurrence and poor prognosis [63, 64].

**Fig. 3:**
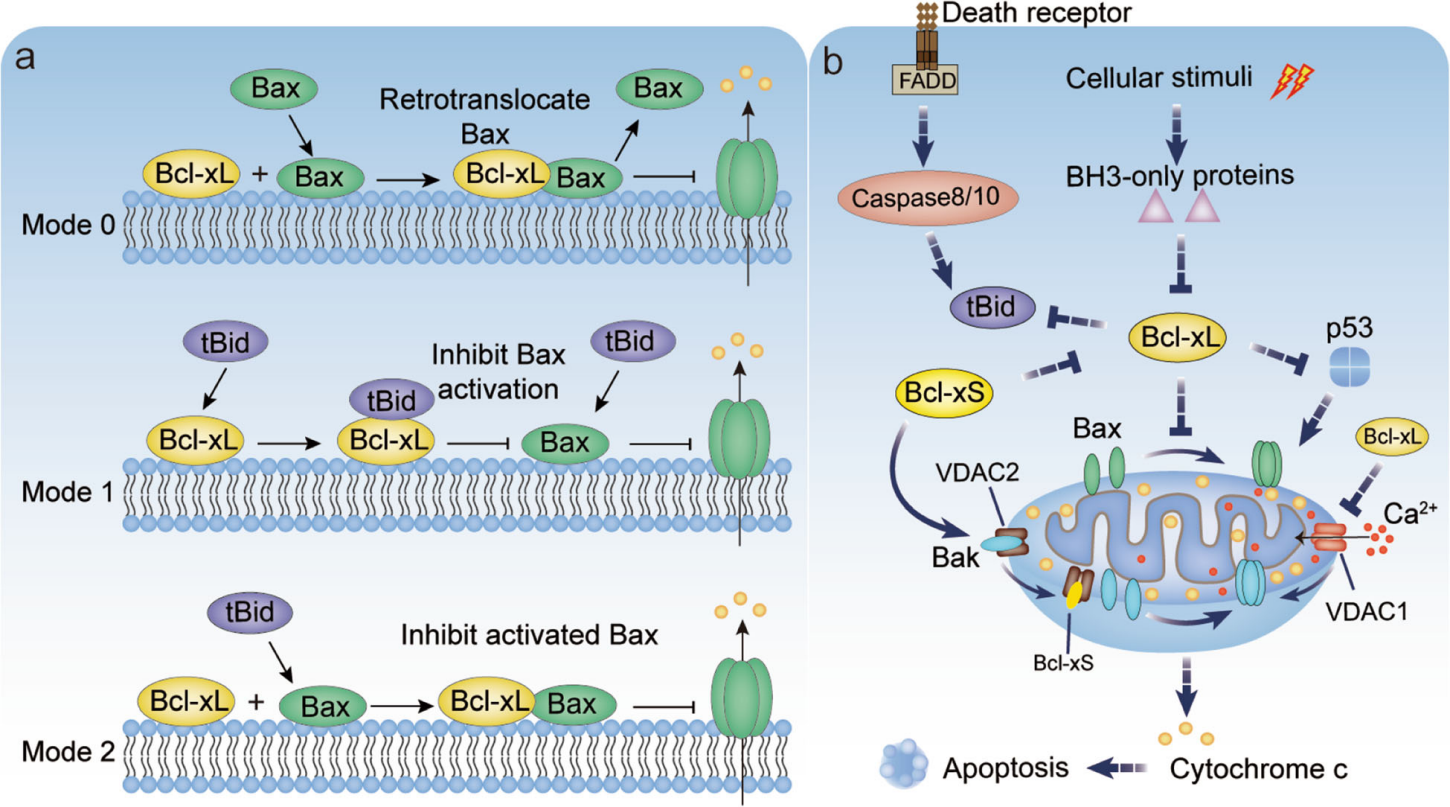
Cell apoptosis regulated by Bcl-x isoforms. **a**. Three modes that had been proposed to explain how Bcl-xL regulate MOMP. Mode 0: Bcl-xL prevented the binding of apoptotic effectors Bax to mitochondrial outer membrane through retrotranslocating Bax from the mitochondria into cytosol constantly. Mode 1: Bcl-xL sequestered BH3-only activators (tBid) to prevent them from binding to and activating Bax. Mode 2: Bcl-xL directly bound to activated Bax to prevent its oligomerization and MOMP. **b**. Cell apoptosis pathways regulated by Bcl-xL and Bcl-xS

Alternative splicing of Bcl-x pre-mRNA is one of the earliest oncogenic splicing events critical for apoptotic responses of cancer cells. The elevated level of Bcl-xL caused by aberrant splicing has been revealed in a multitude of human cancers and is considered to be a powerful driving force for cell apoptotic resistance (Table 2) [1, 78]. Instead, cells with highly expressed Bcl-xS were more sensitive to apoptosis stimuli [25]. Structural features enabled Bcl-xL to bind to its natural ligands, such as pro-apoptotic Bcl-2 family members that respond to a variety of cellular stimuli [64]. This process had been well documented by the tertiary structure of Bcl-xL/BH3 peptides, that pro-apoptotic BH3 peptide bound to the hydrophobic groove of Bcl-xL via hydrophobic and electrostatic interactions [88]. In general, Bcl-xL distributed on the intracellular membrane appeared to regulate apoptosis mainly by three modes. In mode 0, Bcl-xL could prevent the binding of apoptotic effectors Bax to mitochondrial outer membrane through retrotranslocating Bax from the mitochondria into cytosol constantly (Fig. 2a) [89]. In mode 1, Bcl-xL could sequester BH3-only activators (For example Bid truncated in the death receptor-mediated pathways (Fig. 3b)) to prevent them from binding to and activating Bax (Fig. 3a). In mode 2, Bcl-xL was suggested to directly bind to activated Bax to prevent its oligomerization and pore formation, which prevented the release of caspase activator from mitochondrial outer membrane (Fig. 3a) [90]. However, Bcl-xL sequestration could be derepressed by sensitizer BH3 only proteins (For example Bad), which then induced activation of Bax and MOMP (Fig. 3b) [90]. Bogner *et al. s*uggested that the allosteric regulation by Bcl-xL complexes might play an important role in this process [91]. Moreover, Bcl-xL was suggested to inhibit a weak Bax activation and apoptotic signal via directly sequestrating active cytosolic p53 induced by damage stimuli (Fig. 3b) [92]. The above demonstrated that apoptosis decision of cells was dependent on the relative abundance of Bcl-xL and its pro-apoptotic counterparts [64, 90]. In addition to the already established roles in mitochondrial apoptotic pathways, Bcl-xL was proved to function as an inhibitor of VDAC1 to prevent apoptosis induced by excessive Ca^2+^ transferred from endoplasmic reticulum to mitochondria (Fig. 3b) [93]. Thus it is imperative to investigate the in-depth mechanism that Bcl-xL used to coordinate apoptotic signals from multiple pathways and ultimately, form an integrated perspective. Compared to Bcl-xL, the short isoform Bcl-xS was reported as a negative regulator of survival because it could inhibit the function of Bcl-xL by forming heterodimers with Bcl-xL through the BH3 domain, or disrupting the VDAC2-Bak complex to cause the release of Bak and activation of MOMP (Fig. 3b) [94, 95]. Furthermore, Bcl-xS induced activation of Bak and promoted apoptosis through apoptosome-dependent and independent pathways [96]. Therefore, the antagonistic roles played by Bcl-x isoforms were critical for cell fate decision. Alternative splicing regulation of Bcl-x to promote Bcl-xS but inhibit Bcl-xL splicing could act as a tumour suppression strategy. For instance, Src family kinase Fyn was found to decrease the phosphorylation of Sam68 but regulate hnRNPA2/B1 expression, which synergistically promoted the splicing of Bcl-xL and inhibited apoptosis of pancreatic cancer cells. Fyn inhibition down-regulated hnRNPA2/B1 expression and increased Bcl-xS splicing [37]. In addition, study on human liver fibrosis suggested that Bcl-xL was preferentially spliced in human hepatic stellate cells and consistent with apoptosis resistance of HSCs. Antisense oligonucleotides inhibiting Bcl-xL splicing induced HSC cell apoptosis [97]. Interestingly, the IE86 gene of human cytomegalovirus was found to inhibit apoptosis and promote proliferation of glioma cells by enhancing the favor splicing of Bcl-xL mediated by hnRNPA2/B1 [98]. Thus, the favored use of 5’PSS splice site in Bcl-x pre-mRNA contributes to the escape of cancer cells from intrinsic programmed apoptosis.

**Table 2:** Aberrant Bcl-x splicing in cancers and its clinical application

	Cancer type	Bcl-xL/S	Function	Ref
Apoptosis	Hepatocellular Carcinomas	Bcl-xL↑	Inhibit apoptosis initiated by cellular stimuli	[65]
	Colorectal cancer (CRC)	Bcl-xL↑	Drive tumourigenesis and progression.	[66]
	Breast cancer	Bcl-xL↑	Suppress BETi-induced apoptosis.	[67]
	Meningioma	Bcl-xL↑	Contribute to apoptosis induced by Dovitinib.	[68]
Malignancy	Gastric cancer	Bcl-xL↑	Associated with high Beclin1 expression.	[69]
	Tongue Carcinoma	Bcl-xL↑	Related to the degree of differentiation.	[70]
	Hodgkin lymphoma	Bcl-xL↑	Consistent with the severity of patients.	[71]
	Myeloproliferative neoplasms	Bcl-xL↑	Progressively over-expressed.	[72]
	Lymphomas	Bcl-X_S/L_↓	Expressed by malignant cells.	[73]
	Wilms' tumours	Bcl-X_S/L_↓	Negatively correlated with tumour stage.	[74]
	Endometrial carcinoma	Bcl-X_S/L_↓	Correlated with pathological grading.	[75]
Metastasis	Pancreatic cancer	Bcl-xL↑	Promote metastasis	[76]
	Glioblastoma	Bcl-xL↑	Promote cell migration, invasion, angiogenesis and stemness.	[77]
	Melanoma	Bcl-xL↑		[77]
Drug-resistance	Chondrosarcoma	Bcl-xL↑	Confer resistance to chemotherapy.	[78]
	Ewing sarcoma	Bcl-xL↑	Resistant to olaparib.	[79]
	Ovarian carcinoma (OC)	Bcl-xL↑	Confer resistance to chemotherapy.	[80]
	Hepatocellular carcinoma	Bcl-xL↑	Chemoresistance and poor prognosis.	[81]
	Urothelial Carcinoma	Bcl-xL↑	Effectively inhibited cisplatin-resistant UCs.	[82]
Radiation	laryngeal cancer	Bcl-xL↑	Associated with radioresistant.	[83]
	Non-small cell lung cancer	Bcl-xL↑	Enhance irradiation resistance.	[84]
	Prostate cancer	Bcl-xL↑	Enhance survival to cells exposured to IR.	[85]
	Osteosarcoma	Bcl-xL↑	Enhance irradiation resistance.	[86]
	Malignant pleural mesothelioma	Bcl-xL↑	Negatively associated with radiosensitivity.	[87]

#### Autophagy

**Fig. 4:**
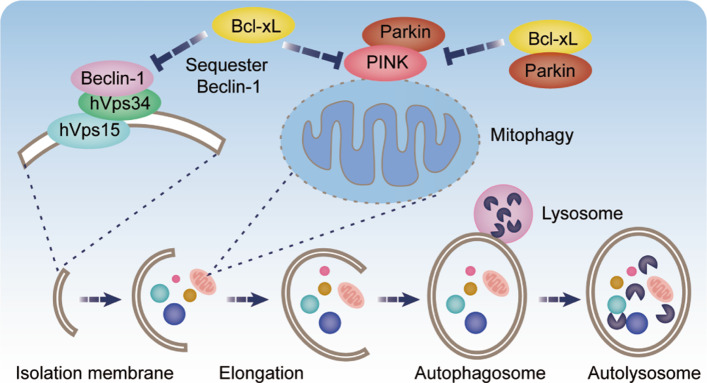
Cell autophagy mediated by Bcl-xL. Bcl-xL inhibited initial steps of autophagy by interacting with the core regulators of autophagy Beclin-1, which disrupted the hVps34–Beclin-1 complex and limited its ability to stimulate autophagosome formation. Bcl-xL also could inhibit PINK1/Parkin-dependent mitophagy through directly interacting with PINK1 and Parkin to inhibit the translocation of Parkin from cytoplasm into mitochondria

In addition to apoptosis, the long isoform Bcl-xL also had been suggested to be involved in autophagy, which was an evolutionarily conserved pathway and played a double-edged role in tumour progression [84]. A recent study explored that Bcl-xL could inhibit PINK1/Parkin-dependent mitophagy through directly interacting with Parkin and PINK1 to inhibit the translocation of Parkin from cytoplasm into mitochondria (Fig. 4) [99]. More importantly, Bcl-xL was identified to hinder macro-autophagy mediated by class III PI3K pathway through direct interactions with Beclin-1, a new BH-3 only protein that is essential to regulate the initial of autophagy [100, 101]. Low expressed Beclin-1 but highly expressed Bcl-xL is consistent with malignant phenotype and poor prognosis of cancer [69, 102]. Bcl-xL physically interacted with BH3 domain of Beclin-1 and disrupted hVps34–Beclin-1 complex which stimulated autophagosomes formation (Fig. 4) [103, 104]. BH3 mimetics and overexpressed BH3-only proteins could displace Beclin-1 from Bcl-xL and stimulate autophagy. Intriguingly, Beclin-1 was reported to induce apoptosis of glioblastoma cells through binding to Bcl-xL [105], whereas another research suggested that heterooligomers formed by Bcl-xL and Beclin-1 could maintain full anti-apoptotic function in HeLa cells induced by staurosporine [106]. These results support the model that direct interactions between Bcl-xL and Beclin-1. However, Lindqvist LM *et al.* suggested that Bcl-xL had no measurable effect on autophagy in the absence of Bax/Bak [107]. When Bax/Bak were present, inhibiting the pro-survival Bcl-2 family members stimulated autophagy and correlated with increased cell death, suggesting that inhibition of Bcl-xL on autophagy was an indirect effect generated from apoptosis inhibition by a yet unknow mechanism. In summary, the possible relevance between apoptosis and autophagy in the process of cell death mediated by Bcl-xL includes:(1) Bcl-xL physically interacts with Beclin-1 to regulate apoptosis and autophagy synergistically or antagonisticly; (2) Bcl-xL does not bind to Beclin 1 but instead regulate autophagy by inhibiting Bax/Bak mediated apoptosis [3]. Thus, further research is required to determin the crosstalk between apoptosis and autophagy mediated by Bcl-xL and other Bcl-2 family proteins, which is of great significance for maintaining the overall cell fates.

#### Invasion and metastasis

Bcl-xL had been suggested to contribute to invasion and metastasis in multiple cancer types. After knocking Bcl-xL, the invasive and metastatic phenotype of CRC cells were reduced but did not cause spontaneous cell death [108, 109]. Studies of oral tongue cancer and breast cancer found that Bcl-xL expression was significantly high in metastasis tissue [4, 70] . In transformed human mammary epithelial cells, Bcl-xL directly interacted with RAS to modulate RAS signaling through BH4 domain, which was critical for RAS induced stemness and cancer initiating cell phenotype [110]. In addition, overexpressed Bcl-xL in human melanoma was found to promote vasculogenic structures through CXCL8/CXCR2 pathway. Meanwhile, the increased cancer stem cell markers associated with stemness and aggression of tumour cells ( for example HIF-1α, NANOG, OCT4, BMI1, and SOX2) were also observed [111]. Notably, a recent study of Bcl-xL with defection in apoptosis suggested that anti-apoptotic function of Bcl-xL might be separated from its roles in the motility of cancer cells. For example, Choi *et al*. [76] showed that Bcl-xL defected in anti-apoptotic function promoted epithelial-mesenchymal transition (EMT), migration, invasion, as well as stemness of panNET and breast cancer cells. Additionally, Bessou *et al*. [112] suggested that the complex formed by BH4 domain of Bcl-xL and VDAC1 acted on MOMP to increase ROS in mitochondrial electron transport chain and inhibit the absorption of Ca^2+^, thereby promoting migration and metastasis of breast cancer cells independently of apoptotic activity. How Bcl-xL regulates the invasion and metastasis of cancer cells independently of apoptosis still needs further exploration.

#### Anti-tumor immunity

Anti-apoptotic Bcl-xL has been demonstrated to play a key role in the survival of immune cells and immune responses. Grillot *et al.* has reported that Bcl-xL is highly expressed in CD4+ CD8+ thymocytes which exhibited increased viability in *vitro* [113]. Enforced Bcl-xL expression could also restore the development of splenic B Lymphocyte [114]. In addition, Bcl-xL was proved to promote survival of effector T cells and antigen-bearing dendritic cells [115, 116]. Regulatory T cells showed enhanced suppressive capacity through increasing Bcl-xL expression, which provide a new strategy for treatment of tumours through remodelling regulatory T cells [117]. Surprisingly, Bcl-xL was demonstrated to protect tumour cells from Natural Killer cells-mediated suppression and therefore exerted tumour-progressive activity [118, 119]. However, Andersen *et al.* suggested highly expressed Bcl-xL of cancer cells were the common target recognized by specific T cells [120]. They also speculated that immune responses against apoptosis inhibitors like Bcl-xL might represent a general feature in cancer [120]. Taken together, there is a complex effect of Bcl-xL expression on anti-tumour immune response. It is of great significance to identify the role of overexpressed Bcl-xL in immune escape of tumor cells.

### Clinical impaction of Bcl-x splicing in cancer

#### Chemotherapy

Tolerability generated during chemotherapy such as apoptosis escape and Epithelial mesenchymal transformation (EMT), results in poor prognosis and is currently impeding the success of targeted therapies in cancer treatment [121, 122]. Plenty of evidence suggested that Bcl-xL-dependent apoptotic inhibition was the main reason that promoted chemotherapy resistance in tumours in *vitro* and *vivo* (Table 2) [123, 124]. A study on breast cancer showed that cells passed through EMT obtained therapeutic resistance by upregulating Bcl-xL transcripts. However, apparent apoptotic resistance was removed after deleting Bcl-xL [4]. Bcl-xL was also found to mediate doxorubicin resistance of breast cancer through the Ring finger protein 6/Estrogen receptor α/Bcl-xL pathway [125]. Inhibiting Bcl-xL expression in breast cancer cells enhanced the cytotoxicity and apoptosis induced by T-DM1 [126]. Additionally, increased CXCR4 expression in ovarian cancer induced cisplatin resistance through promoting Bcl-xL/S [123]. Upregulated Bcl-xL expression was also found to be involved in resistance to therapy targeting Bcl-2 in mantle-cell lymphoma and Acute Myelocytic Leukemia [57, 127]. Regarding melanoma, it has been demonstrated that forced expression of ectopic Bcl-xL converted drug-sensitive cell lines into drug-resistant ones [128]. However, *vivo*-Morpholino (vMO) antisense oligomers that used to upregulate Bcl-xS expression but decrease Bcl-xL in chronic myeloid leukemia (CML) increased growth inhibition and apoptotic sensitivity of imatinib mesylate-resistant CML cells [5]. Similarly, overexpressed Bcl-xS in human breast carcinoma cells induced a remarkable increase in sensitivity to chemotherapy agents, but did not affect cell viability by itself [129].

#### Radiotherapy

The splicing favor of Bcl-xL contributed to long-term radiotherapy resistance (Table 2). Clinical data showed that Bcl-xL was expressed by about 91% of laryngeal cancer patients resistant to radiotherapy, suggesting a critical function of Bcl-xL in radiotherapy [83]. Streffer *et al.* [130, 131] found that glioma cell lines with high Bcl-xL expression had higher ED50 (2.9 ± 0.8Gy) than cell lines with lower Bcl-xL. However, no association with radiosensitivity was observed for the expression levels of Bcl-xS. Highly expressed Bcl-xL was also found to cause radiation resistance of osteosarcoma cells with both low and high metastasis level, and Bcl-xL downregulation could significantly enhance radiation cytotoxicity of osteosarcoma cells [86]. Moreover, inhibiting the expression level of Bcl-xL were suggested to reverse radio-resistance and regulate radiation-induced apoptosis of mesothelioma, breast cancer, prostate cancer, colorectal cancer as well as non-small cell lung cancer [84, 87, 132, 133]. In addition to therapeutic effects, irradiation was well known to induce increased invasiveness and metastasis of cancer cells. Ho *et al.* demonstrated that the expression of Bcl-x was elevated after irradiation, which promoted the malignant actions of lung cancer cells [134]. A recent study also suggested that upregulated Bcl-xL induced invasion of cancer cells that underwent sublethal doses of irradiation by stimulating respiratory complex I and increasing additional ROS production, which might be involved in the local recurrence or distal metastasis of somne patients after radiotherapy [135]. Interestingly, the expression of Bcl-xL could enhance energy metabolism and prevent oxidative stress, which might be involved in the alleviation of mitochondrial oxidative stress induced by radiation [136]. In addition, inhibition targeting Bcl-xL/2 had been found to reverse the pulmonary fibrosis induced by ionizing radiation [137]. These results provided a wealth of evidence that inhibition the endogenous expression of Bcl-xL might promote both radiation sensitization and radiation protection. However, combination of γ-irradiation and genetic loss but not pharmacological inhibition of Bcl-xL was found to cause fatal kidney damage and secondary anemia in adult mice, and the underlying mechanism remained unclear [138]. This finding demonstrated the protective role of Bcl-xL in adult kidney, which also represents challenges for the radio-sensitization targeting Bcl-xL.

### Strategies modulating Bcl-x splicing in cancer

#### Splice-switching oligonucleotides

**Fig. 5:**
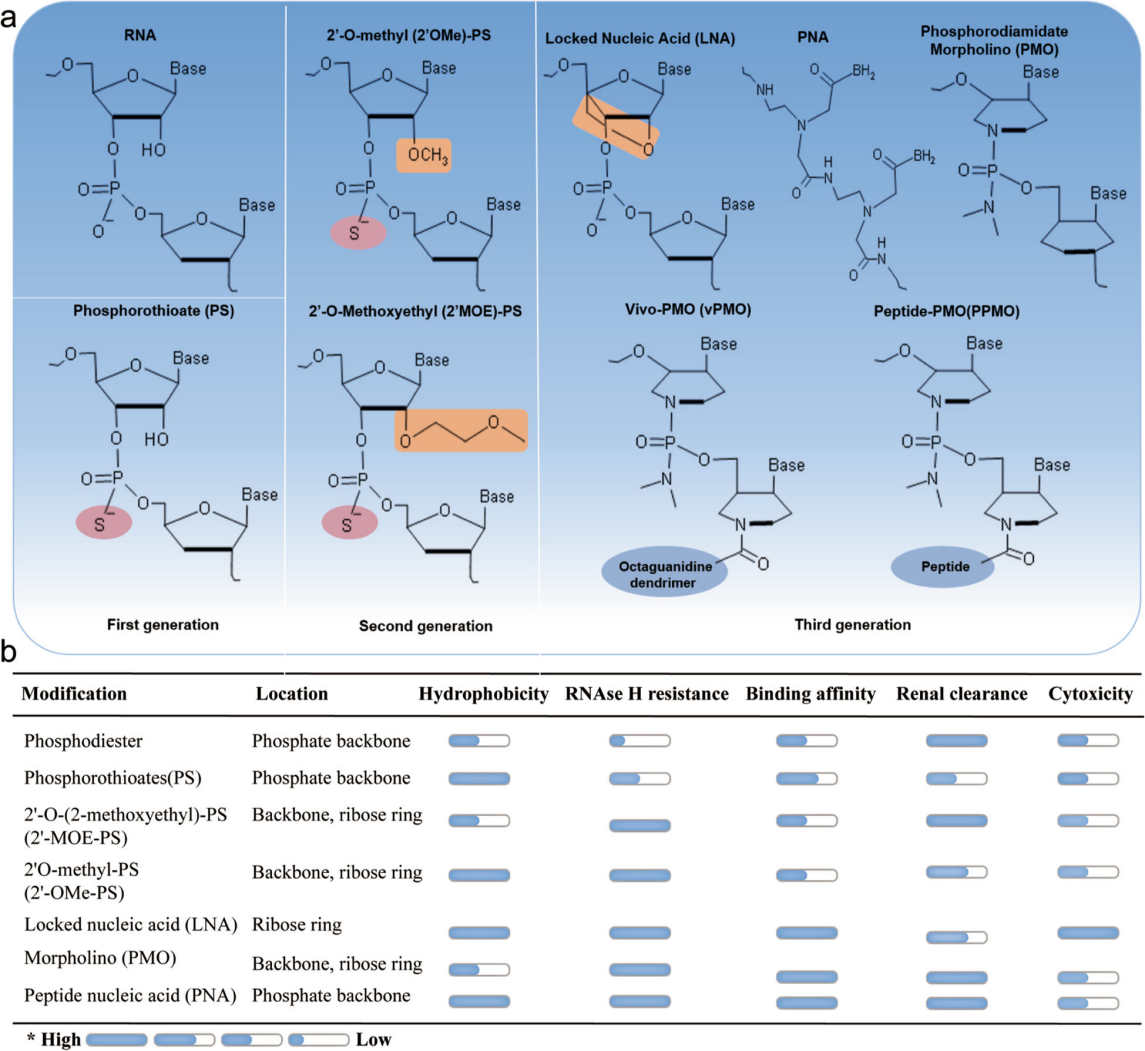
Chemical modifications of splice switching oligonucleotides. **a**. Chemical modifications on phosphate backbone and ribose ring of SSOs. Unmodified RNA is shown for reference. PS, one of the phosphate backbone oxygen atom is replaced by a sulphur atom; 2′-MOE and 2′-OMe, PS-SSOs are often combined with ribose modifications including 2′-O-(2-methoxyethyl) or 2′O-methyl; PMO, charge-neutral nucleic acid, in which the six-membered morpholine ring replaces the five-membered ribose heterocycle; PPMO, positively charged peptides in PPMO dramatically improve intracellular uptake of PMO. VPMO, covalently linking MO to an octaguanidine dendrimer to improve delivery efficacy. LNA, the second and fourth of ribose form a rigid structure by shrinkage. PNA, a pseudo peptide polymer backbone substitutes for the phosphate backbone of RNA. **b**. Properties comparison of the common chemistries of antisense oligonucleotides

**Fig. 6:**
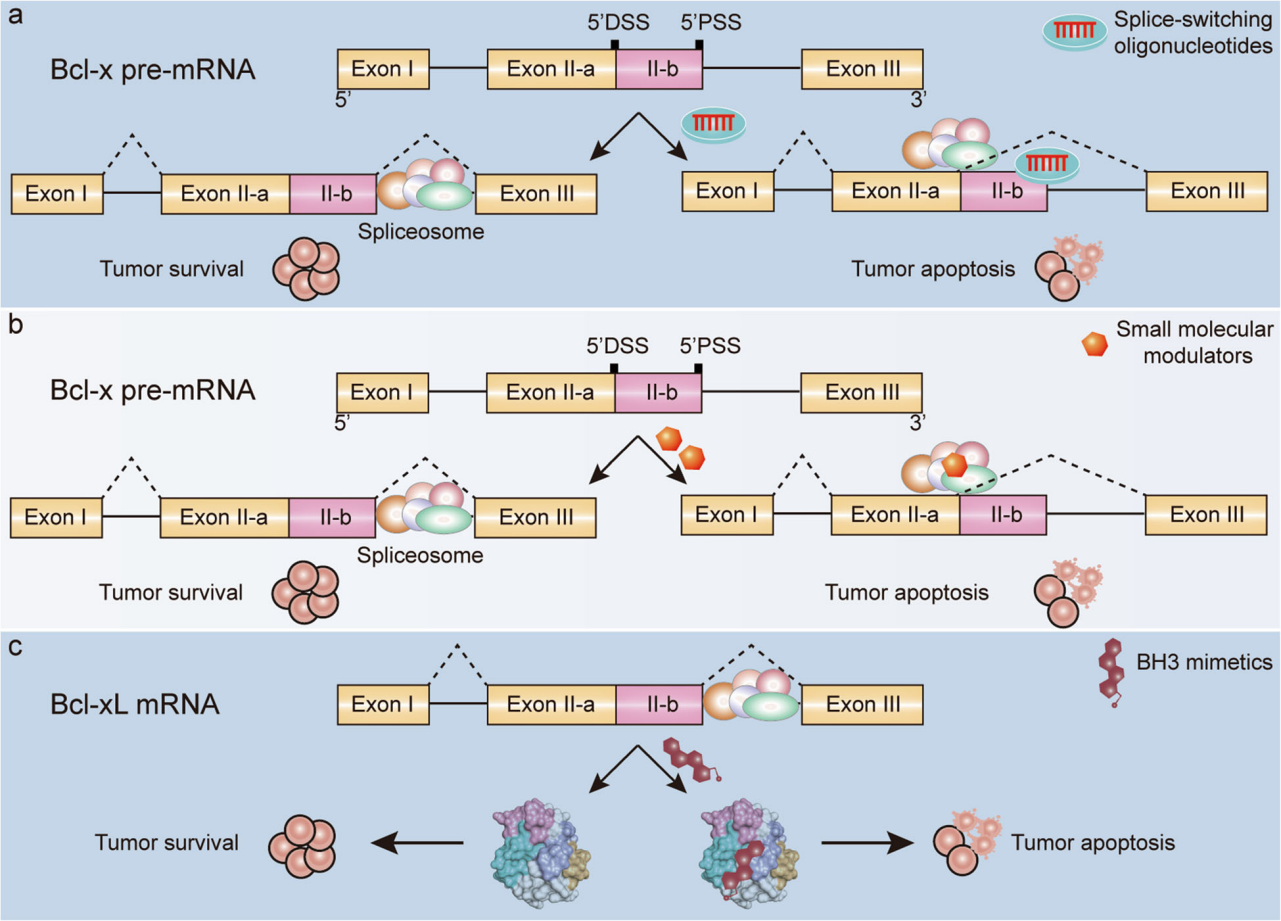
Strategies modulating Bcl-x splicing in cancer. **a**. An SSO that binds to the proximal 5' splice site (5'PSS) prevents binding of spliceosome, leading to a splicing shift to the short isoform Bcl-xS. **b**. a. The small molecular modulators that bind to spliceosomal components affect splice-site accessibility, leading to an inhibition of Bcl-xL splicing. **c**. At the protein level, BH3-mimetics could occupy the hydrophobic pockets of Bcl-Xl, thus blocking their anti-apoptotic activity and resulting in the ignition of apoptosis

SSOs, typically 15-30 nucleotides, is a kind of synthetic, modified, steric block antisense oligonucleotides which have been widely used to disrupt the splicing mode of pre-mRNA through Watson-Crick base pairing. The generated steric hindrance but not degradation of targeted transcripts affected accessibility of splicing factors and visibility of spliceosome, which led to splicing isoforms switching ultimately [139]. Notably, natural oligonucleotides had been proved to be quite ineffective due to their defects such as easy to be degraded, lower affinity, and higher off-target effect. Therefore, various chemical modifications on phosphate backbone or ribose rings of SSOs had been developed to allow for improved stability and binding affinity, meanwhile, reduced cytotoxicity and immunogenicity [139]. Common types of oligonucleotides chemistry have been shown in Fig. 5. Notably, the third generation of antisense oligonucleotides was featured by furanose ring modifications of nucleotides including phosphorodiamidate morpholinos (PMOs), locked nucleic acid and peptide nucleic acid. PMOs was a type of neutrally charged nucleic acid, in which the furanose ring was substituted by a morpholine ring while each unit was bridged with a phosphorodiamidate linkage. PMOs usually needed to be conjugated to cell-penetrating peptides or covalently linked to an octaguanidine dendrimer for efficient delivery due to their rapid renal clearance. To date, PMOs modified SSOs drugs eteplirsen and golodirsen had been approved by the FDA for clinical therapy of Duchenne muscular dystrophy and spinal muscular atrophy, respectively [140, 141]. In addition, for effective SSOs of target genes, the optimized length, sequence constitution, secondary structures, accessibility, as well as thermodynamic properties were all critical factors [92]. Generally, SSOs base-paired to the alternative splice siteof Bcl-x pre-mRNA could block arrival of spliceosome and binding of splicing factors to their target motif, which led to the redirection of splicing favor (Fig. 6a). Bcl-xSSOs could promote apoptosis and drug sensitivity of cancer cells by correcting Bcl-xL splicing to Bcl-xS efficiently [142]. Some Bcl-xSSOs sequences used in preclinical had been summarized in Table 3. Mercatante *et al.* proved that endogenous highly expressed Bcl-xL was positively correlated with cellular response to Bcl-xSSOs induced splice shift [147], which indicated that normal cells with low expressed Bcl-xL might be more resistant to Bcl-xSSOs therapy. 2'-OMe-PS modified Bcl-xSSOs caused a splice shift from Bcl-xL to Bcl-xS and increased apoptosis of prostate cancer, breast cancer, and hepatic stellate cells [97, 147]. In addition, splice redirection of Bcl-x pre-mRNA induced by 2'-MOE modified Bcl-xSSOs in glioma cells and melanoma xenograft models showed pro-apoptotic effect and reduced tumour load respectively [142, 143]. Moreover, vMO modified Bcl-xSSOs was found to correct aberrant splicing of Bcl-x in CML cells and improve therapeutic sensitivity to imatinib mesylate significantly [5, 148]. Therefore, highly expressed Bcl-xL could be reversed by modified Bcl-xSSOs, which allowed the redirection of aberrant splicing and rebalanced the ratio of Bcl-xL/Bcl-xS [6].

**Table 3:** Splice switching oligonucleotids used to modulate Bcl-x pre-mRNA splicing

Cells types	Sequence	Length	Chemistry	Ref
K562	5'-GCTTGGTTCTTACCCAGCCGCCGTT-3'	25 mer	vMO	[5]
Primary HSCs	5'-TGGTTCTTACCCAGCCGCCG-3'	20 mer	2'-OMe-PS	[97]
U87, U251	5'-TGGTTCTTACCCAGCCGCCG-3'	20 mer	2'-MOE-PS	[142]
B16F10	5'-TGGTTCTTACCCAGCCGCCG-3'	20 mer	2'-MOE-PS	[143]
Human RPE	5'-TGGTTCTTACCCAGCCGCCG-3'	20 mer	2'-MOE	[144]
A549	5'-CTGGATCCAAGGCTCTAGGT-3'	20 mer	2'-MOE	[145]
PC-3	5'-ACCCAGCCGCCGUUCUCC-3'	18 mer	2'-OMe-PS	[146]
MCF-7	5'-ACCCAGCCGCCGUUCUCC-3'	18 mer	2'-OMe-PS	[146]
Hela	5'-ACCCAGCCGCCGUUCUCC-3'	18 mer	2'-OMe-PS	[146]

#### Small molecular modulators redirect Bcl-x splicing

A class of natural or synthetic small molecular modulators had been identified to perform anti-tumour activity through inhibiting the activity of targeted splicing factors (Fig. 6b) [149, 150]. These compounds modulated RNA splicing in two ways generally. One way was to directly target pre-mRNA splicing factors. For example, the crude extract of the South African Medicinal Plant (*Cotyledon orbiculata)* could induce a splicing shift from Bcl-xL to Bcl-xS and apoptosis of cancer cells [151]. However, it was interesting that Bcl-x exhibited resistance to splicing perturbation induced by SF3B1 inhibitors E7107. Splicing modulation of E7107 was sensitized after Bcl-xL knockdown, which suggested that Bcl-xL could function as a sensitizing gene or as a biomarker for splicing modulator treatment [152]. The other way was to target kinases that were involved in post-translation modulation of splicing factors. For example, potent protein synthesis inhibitor emetine had been proved to enhance Bcl-xS splicing in a time and dose-dependent manner in multiple cancers, however, this effect could be hindered by protein phosphatase 1 (PP1 )[153]. Alkaloid Homoharringtonine approved for CML treatment by the FDA as well as antihypertensive agent amiloride and its derivatives BS008 were all proved to normalize oncogenic splicing pattern of Bcl-x in cancer cells depending on PP1 activation [154–156]. Intriguingly, Moore *et al.* found that inhibitors of cell cycle factors aurora kinase A (for example ZM447439 and VX-680) could induce endogenous Bcl-xS splicing significantly, revealing a complex alternative splicing network coordinating cell cycle and apoptosis [157]. In general terms, studying the mechanism of splice switch induced by small molecular modulators is essential for splicing therapies and antitumour agent discovery based on splicing correction. Notably, although the effectiveness that small molecules showed in splicing modulation, they usually lacked specificity and caused off-targeted or on-targeted cytotoxicity.

#### BH3 mimetics inhibit Bcl-xL isoform

Selective or multi-targeted BH3 mimetics had been developed to antagonize anti-apoptotic proteins of Bcl-2 family through competitively occupying the hydrophobic pockets and thus blocking their anti-apoptotic activity (Fig. 6c) [158]. Table 4 listed BH3-mimetics targeting anti-apoptotic Bcl-2 family proteins selectively. A-1331852 and WEHI-539 selectively targeted to Bcl-xL were all proved to enhance death signals of cancer cells synergistically with radiation or chemotherapy agents [158, 187]. In addition, compounds DT2216 and XZ424 converted from BH3 mimetics by proteolysis-targeting chimera showed improved anticancer potency and reduced cytotoxicity based on E3 ligase mediated degradation of Bcl-xL [162, 163]. However, use of BH3-mimetics in chronic lymphocytic leukaemia and other solid tumours exhibited on-target and off-target effects of Bcl-xL dependent cells and pathways [158, 188–190]. In addition, efficacy of BH3 mimetics was intensely dependent on the membrane localization of Bcl-xL and the nature of interactions between Bcl-xL and pro-apoptotic proteins, which might contribute to a chemotherapeutic resistance of BH3 mimetics [191]. These are still the obstacles for clinical application of BH3 mimetics. Consequently, optimizing the pharmacological effect and concurrent targets of BH3 mimetics to make them promising therapeutic regimens of cancer has been challenging.

**Table 4:** Clinical application of BH3-mimetics targeting anti-apoptotic Bcl-2 family proteins. (clinicaltrials.gov)

Multiple targets	Compounds	Origin	Stage	Ref
Bcl-xL	A-1155463	Structure-based design.	Preclinical	[159]
	A-1331852	Structure-based design.	Preclinical	[160]
	WEHI-539	Structure- based design	Preclinical	[161]
	DT2216	Proteolysis targeting chimera	Preclinical	[162]
	XZ424	Proteolysis targeting chimera	Preclinical	[163]
	ABBV-155	Structure-based design	Phase I	NCT03595059
Bcl-xL Bcl-2	AZD4320	Structure-based design	Preclinical	[164]
	BM-957	Structure-based design	Preclinical	[165]
	BM-1197	Structure-based design	Preclinical	[166]
	S44563	Structure-based design	Preclinical	[167]
	APG-1252	Structure-based design	Phase I/II	[168]
Bcl-xL Bcl-2 Bcl-w	Ch282-5	Gossypol derivative	Preclinical	[169]
	ABT-737	Synthetic, acylsulfonamide-based	Phase I/II	[170]
	ABT-263 (Navitoclax)	Derivant of ABT-737	Phase I/II/III	[171]
Bcl-xL, Bcl-2, Mcl-1	BH3-M6	Synthetic terphenyl scaffold	Preclinical	[172]
Bcl-xL, Bcl-2, Bcl-w, Mcl-1	TW-37	Benzenesulfonyl derivative of gossypol	Preclinical	[173]
	BI-97C1 (Sabutoclax)	Diastereoisomer of Apogossypol	Preclinical	[174]
	BIM-SAHB	Stapled Bim peptide	Preclinical	[175]
	GX15-070 (Obatoclax)	Synthetic indolyl-dipyrromethene	Phase I/II/III	[176]
	AT-101	(−)-gossypol enantiomer	Phase I/II/III	[177]
Bcl-2	S55746	Structure-based design	Phase I	[178]
	ABT-199 (Venetoclax)	Derivant of ABT-263	Phase I/II/III	[127]
Mcl-1	A-1210477	Structure-based design	Preclinical	[179]
	UMI-77	Structure-based design	Preclinical	[180]
	VU661013	Fragment-based lead generation	Preclinical	[181]
	S63845	Structure-based design	Preclinical	[182]
	AMG176,	Structure-based design	Phase I	[183]
	AZD5991	Structure-based design	Phase I	[184]
	S64315	Fragment-based lead generation	Phase I/II	[182]
Bcl-2, Mcl-1	S1-6	Structure-based design	Preclinical	[185]
	Nap-1	Derivant of S1-6	Preclinical	[186]

## Conclusions

The inactivation or dysfunction of essential genes caused by defective splicing is emerging as a potential driver of cancer development. Therefore, controlling splicing is of great therapeutic benefit. Dysregulated Bcl-x splicing plays a key role in promoting malignant phenotypes of cancers and weakening the toxicity of treatment. Bcl-xL contributed to the invasion, metastasis, and angiogenesis of cancers. On the contrary, Bcl-xS overexpression was suggested to sensitize apoptosis induced by drugs [129]. Bcl-x splicing correction by SSOs and small molecular modulators showed efficiency in apoptosis regulation of cancer cells. However, the on-targeted toxicity to Bcl-xL-dependent cell types posed challenges to the exploitation and delivery of splicing modulation drugs, which was expected to be addressed by the breakthrough of drug chemistry and carrier system [6]. In addition, the inhibitors of specific splicing factors for Bcl-x splicing correction are needed to be identified. Generally, induction of the balanced ratio of Bcl-xL/Bcl-xS has been shown anti-tumour activity by targeting multiple hallmarks of tumour, but it is still imperative that we understood this biomolecule. It is still unknown what is the intracellular mechanism that induced the preferred splicing of long isoform Bcl-xL. In addition, to discover the interplay of apoptosis and autophagy regulated by Bcl-xL means great significance to Bcl-xL targeted therapy. Moreover, whether the diverse domains of Bcl-xL execute biological functions independently and how does membrane localization affect its biological function in vivo remains unknown. Little is known about the biological function of Bcl-xS beyond its canonical function of lowering apoptosis threshold. In addition to Bcl-x, the anti-apoptotic family members including Bcl-2, Mcl-1 and Bcl-w also have a variety of splice isoforms, however, the elaborate coordination of biological roles played by multiple splice isoforms of Bcl-2 family members is unclear. Thus, much more remains to be researched about this gene in the future.

## Data Availability

Not applicable

## Abbreviations

Bcl-x: Bcl-extra
Bcl-2: B-cell lymphoma 2
SSOs: Splicing-switch oligonucleotide
5' PSS: Proximal 5' splice site
5' DSS: Distal 5' splice site
TM: Transmembrane
IDR: Intrinsically disordered region
MOMP: Mitochondrial outer membrane permeabilization
ROS: Reactive oxygen species
PKC: Protein kinase C inhibitor
GM-CSF: Granulocyte-macrophage colony-stimulating factor
SR: Serine/arginine-rich
hnRNPs: Heterogeneous nuclear ribonucleoproteins
EMT: Epithelial-mesenchymal transition
CML: Chronic myeloid leukaemia
vMO: Vivo-Morpholino
PS: Phosphorothiate
2'-OMe: 2'-O-methyl
2'-MOE: 2'-O-methoxyethyl
ESS: Exonic splicing silencer
ESE: Exonic splicing enhancer
PP1: Protein phosphatase 1
